# Mechanism of Resistance Development in *E. coli* against TCAT, a Trimethoprim-Based Photoswitchable Antibiotic

**DOI:** 10.3390/ph14050392

**Published:** 2021-04-21

**Authors:** Anna I. Lauxen, Piermichele Kobauri, Michael Wegener, Mickel J. Hansen, Nicole S. Galenkamp, Giovanni Maglia, Wiktor Szymanski, Ben L. Feringa, Oscar P. Kuipers

**Affiliations:** 1 Department of Molecular Genetics, University of Groningen, Nijenborgh 7, 9747 AG Groningen, The Netherlands; annalauxen@gmail.com; 2 Stratingh Institute for Chemistry, University of Groningen, Nijenborgh 4, 9747 AG Groningen, The Netherlands; p.kobauri@rug.nl (P.K.); mrwegener@outlook.com (M.W.); mickeljhansen@gmail.com (M.J.H.); 3 Groningen Biomolecular Science & Biotechnology Institute, University of Groningen, Nijenborg 4, 9747 AG Groningen, The Netherlands; n.s.galenkamp@rug.nl (N.S.G.); g.maglia@rug.nl (G.M.); 4 Medical Imaging Center, University Medical Center Groningen, University of Groningen, Hanzeplein 1, 9713 GZ Groningen, The Netherlands

**Keywords:** photoswitchable antibiotic, trimethoprim, TCAT, resistance mechanism, *E. coli*, TolC, exopolysaccharides

## Abstract

During the last decades, a continuous rise of multi-drug resistant pathogens has threatened antibiotic efficacy. To tackle this key challenge, novel antimicrobial therapies are needed with increased specificity for the site of infection. Photopharmacology could enable such specificity by allowing for the control of antibiotic activity with light, as exemplified by *trans*/*cis*-tetra-*ortho*-chloroazobenzene-trimethoprim (TCAT) conjugates. Resistance development against the on (irradiated, TCATa) and off (thermally adapted, TCATd) states of TCAT were compared to that of trimethoprim (TMP) in *Escherichia coli* mutant strain CS1562. Genomics and transcriptomics were used to explore the acquired resistance. Although TCAT shows TMP-like dihydrofolate reductase (DHFR) inhibition in vitro, transcriptome analyses show different responses in acquired resistance. Resistance against TCATa (on) relies on the production of exopolysaccharides and overexpression of TolC. While resistance against TCATd (off) follows a slightly different gene expression profile, both indicate hampering the entrance of the molecule into the cell. Conversely, resistance against TMP is based on alterations in cell metabolism towards a more persister-like phenotype, as well as alteration of expression levels of enzymes involved in the folate biosynthesis. This study provides a deeper understanding of the development of new therapeutic strategies and the consequences on resistance development against photopharmacological drugs.

## 1. Introduction

The rapid emergence of resistant bacteria poses an increasingly growing problem to human society. This rise in antimicrobial resistance (AMR) has been attributed to the misuse and overuse of antibiotics, as well as accumulation of antibiotics in the environment and the lack of new drug development by the pharmaceutical industry [1,2]. Prolonged exposure to non-lethal concentrations of antibiotics can drive bacteria towards resistance development. AMR can be acquired by bacteria through mutations, horizontal gene transfer and by altering gene expression. It has been reported that resistance can be reversible and genetically acquired resistance is relatively more stable than resistance acquired through altered gene expression, while both types of resistance can be lost once the selective pressure is removed [3,4]. 

One of the major causes of AMR development is the off-target activity of broad-spectrum antibiotics, both in the patient’s intestinal microbiota [5] and in the environment [6]. Photopharmacology [7,8,9] could aid in the fight against AMR by offering a possible solution to the lack of selectivity in antibacterial treatments. This research field, which has emerged at the interface of medicinal chemistry and photochemistry, uses light to control the bioactivity of drugs with high spatiotemporal precision and excellent bio-orthogonality [7,8,9].

Photocontrol of antibiotic activity has been achieved by the modification of drug structures with molecular photoswitches [10] such as azobenzene [11,12], spiropyran [13] and diarylethene [14,15], enabling the reversible isomerization between two states by means of light. In the prototypical case of azobenzene, irradiation with suitable wavelengths results in photoisomerization of the thermodynamically stable *trans* isomer to the metastable *cis* isomer, which re-isomerizes to *trans* over time or can be switched back with a light of a different wavelength [16]. Due to the pronounced difference in shape and polarity of those isomers, azobenzene photoswitch has become the most used tool of photopharmacology [10]. Their application is further supported by their well-understood photoswitching process [16], which typically does not involve the formation of reactive oxygen species as a side reaction.

**Figure 1:**
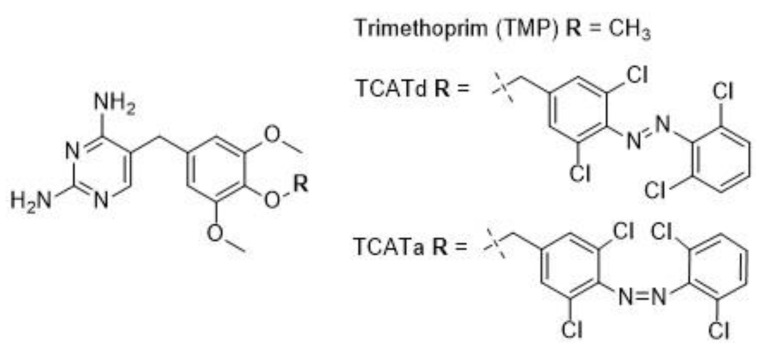
Structural formulas for TMP, TCATd and TCATa. The design of TCATa and TCATd is based on TMP; the difference in rest group (**R**) for each compound is shown in detail.

For useful applications in light-controlled antimicrobial therapy, the thermally stable isomer of the drug should have low or no antibacterial activity, whereas the metastable isomer should be more potent [12]. Hence, two therapeutic scenarios can be envisioned for photoswitchable antibiotics, depending on the wavelengths that can be used for their activation [12]. If the drug is responsive to the cytotoxic UV light [11,13], irradiation before administration would yield a metastable active antibiotic that can reduce the environmental build-up of bioactive substance by losing its activity over time. If the drug is responsive to visible or near-IR light [17] (i.e., the so-called therapeutic window [18]), localized activation at the site of infection would also diminish off-target side-effects, thus further lowering the selective pressure exerted by the antimicrobial agent. In an effort towards the latter scenario, systematic modifications of the broad-spectrum antibiotic trimethoprim (TMP) led to a tetra-*ortho*-chloroazobenzene-TMP (TCAT) conjugate [12], which could be activated in situ with red light (λ = 652 nm) in the presence of bacteria. This visible-light-photoswitchable antibiotic showed a very promising >8-fold increase in antibacterial activity against *E. coli* upon irradiation (TCATa) [12], and it is referred to as the on-state of the conjugate. The thermodynamically stable *trans* isomer (TCATd) is less active and therefore referred to as the off-state of the conjugate. The structures of TMP, TCATd and TCATa are given in Figure 1. The implications for AMR development against both states of the conjugate remain to be investigated.

The design of TCAT was based on TMP, which was first approved in 1979 as a standalone drug and has since then been commonly used in the treatment of urinary tract infections [19]. TMP acts by selectively inhibiting dihydrofolate reductase (DHRF) and thereby blocking the reduction of dihydrofolate (DHF) to tetrahydrofolate (THF), the active form of folate and an essential cofactor in the biosynthesis of proteins and nucleic acids. The critical metabolic pathway inhibited by TMP is thymine synthesis [20,21,22]. Shortly after the introduction of TMP, resistance was already reported in several strains [22]. Resistance against TMP has been intensively studied and can be caused through several different mechanisms, including alteration of the permeability barrier and/or efflux pumps, mutational and regulation changes in the target enzyme and acquired resistance by drug-resistant target enzymes, often acquired through horizontal gene transfer [19]. To gain insight into the clinical potential of new photoswitchable analogs of existing antibiotics, the mechanism of action and selective pressure towards resistance development of TCAT was compared to that of TMP in *Escherichia coli* (CS1562). Using genomics and transcriptomics on samples of differentially treated cells, the genetic mechanism underlying the acquired resistance was studied.

## 2. Results

### 2.1. DHFR Inhibition Assay

**Figure 2:**
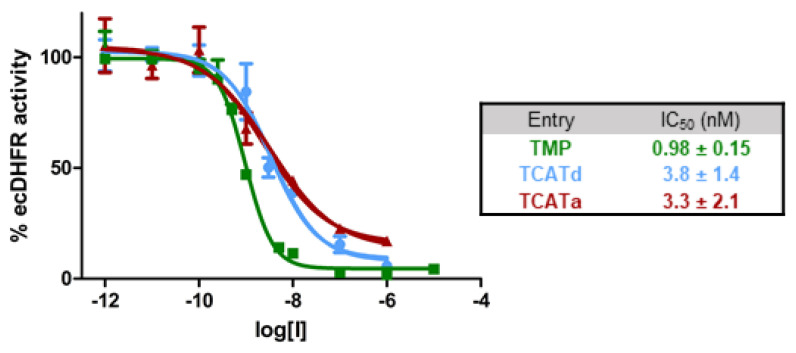
Dose–response curves for TCATd (blue, >99% *trans*-TCAT), TCATa (red, 87:13 *cis*:*trans* distribution) and TMP (green) against *E. coli* DHFR.

In our previous study, the antibiotic activity of TCAT was evaluated exclusively by recording and comparing bacterial growth curves [12]. To confirm that the in vitro mechanism of action of TMP is maintained by our photoswitchable analog, DHFR inhibition was assessed via a colorimetric assay (see Materials and Methods). The potency of the thermally adapted sample of TCAT (IC_50_ = 3.8 ± 1.4 nM) did not change significantly upon irradiation (IC_50_ = 3.3 ± 2.1 nM), while both states exhibited a slight decreased activity when compared to TMP (IC_50_ = 0.98 ± 0.15 nM), as illustrated by the dose–response curves in Figure 2. Although no significant difference in activity was observed between the photoisomers, TCAT conjugates were shown to be highly potent DHFR inhibitors, indicating that the photopharmacological modification of TMP did not alter the in vitro affinity for the original target.

### 2.2. Isolation of Resistant Variants

TMP and TCAT resistant variants were obtained as described in the Materials and Methods. A TMP resistant variant of *E. coli* CS1562 was obtained that could grow in the presence of 1.4 µM TMP, which is 25 times the MIC value of the CS1562 parent strain (Table 1). Interestingly, previous research has found Enterobacteriaceae to be resistant to concentrations of TMP exceeding 3400 µM or a MIC value of the parent strain that is increased by >1000-fold, suggesting the limit of resistance has not yet been reached [19,21]. A resistant variant against TCATa was obtained, growing at 49.3 µM, a 10-fold increased MIC value compared to the parent strain. Resistance to TCATd could not be increased significantly due to solubility limitations and resulted in a MIC value of 98.5 µM, only 1.2 times higher than the parent strain. To date, resistance to TCATa and TCATd molecules has not been described yet.

### 2.3. Genomic Mutations Cannot Fully Explain the Acquired Resistance

Genomic DNA of all resistant strains as well as the propagated parent strain, which served as a control, was isolated and sent for whole genome sequencing. A genome assembly was performed, and the assembled genomes were compared to the control to exclude effects resulting from repeated inoculation only.

Interestingly, all resistant variants shared a subset of intergenic mutations compared to the control, which can roughly be divided into two groups. The first group contains two mutations between the genes encoding tRNA-Val and tRNA-Lys (Δ2 bp, +2/−2) (Δ1 bp, +3/−2). The second group contains five intergenic mutations that are all located between *chaA* and *kdsA* (Table 2). These genes encode the sodium-potassium/proton antiporter ChaA and a 3-deoxy-8-phospooctulonate synthase. The resistant variants also share an intergenic mutation between the *aqpZ* and *lysO* genes (G→A, +202/−293), which encode the aquaporin Z and L-lysine exporter LysO, respectively, and a group of intergenic mutations that are not in the vicinity of any neighboring genes. The intergenic nature of these mutations might provoke the thought that they do not contribute to the gained resistance; however, previous studies have indicated that intergenic mutations occur frequently during antibiotic resistance development and might play a role in the evolution of this pathogenic phenotype by changing expression levels or gene regulation [23,24].

The TCATa^R^ isolate contained two additional mutations, a 1bp deletion in a major facilitator superfamily (MFS) transporter (87/1257 nt) and a large 2934bp deletion encompassing *tetR(A)*, *tet(A)* and *tetC* that are excised from the *tolC* gene (Table 3). The parent strain is an *E. coli* K12 CS180 derivative that contains a stable transposon (*tolC6::mini-Tn10*) inside the *tolC* gene locus [25]. In this isolate, a clean excision of the transposon occurred, thereby restoring the *tolC* locus and subsequently its protein function. TolC is the common outer membrane channel of efflux systems that pump out a large variety of compounds, including several antibiotics. TolC deficient cells show a decreased growth rate, altered morphology and are subject to membrane stress [26]. Deletion of this transposon indicates TolC might play an important role in acquiring resistance against TCATa.

### 2.4. Transcriptomics Reveals Different Approaches towards Resistance Development between Isolates

In addition to the genomic evaluation of these strains, RNA-seq was performed to gain a more in-depth insight in the acquired resistance. Raw sequence reads were mapped using Bowtie2 and count values were taken as an input for the T-REx analysis pipeline, as previously described [27,28]. To find differential gene expression, all resistant isolates were compared to the control (propagated parent strain), as explained by the contrasts file (Table 4).

**Figure 3:**
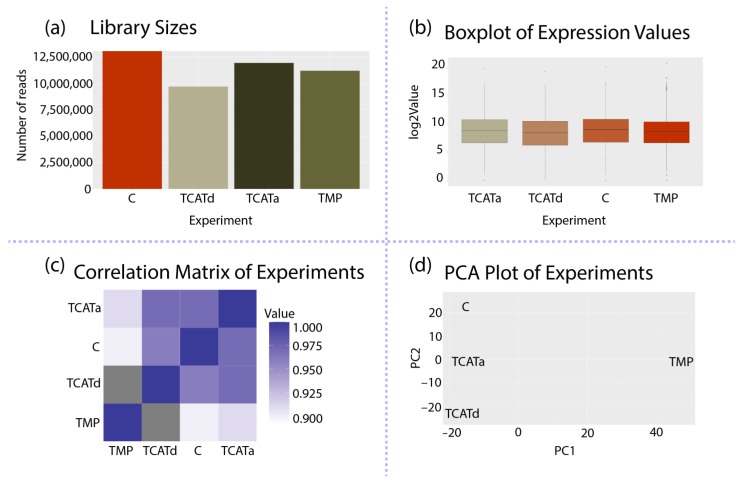
Results of global analysis of the dataset with T-REx: (**a**) library sizes; (**b**) box plot of normalized signals for each sample; (**c**) correlation matrix of the experiments; and (**d**) Principal Component Analysis (PCA) of the experiments. Experiment names are plotted as vectors (points).

Global analysis showed that the library sizes and signal distributions were comparable for all samples (Figure 3). Principal Component Analysis (PCA) of the experiments revealed that the control and the TCATa^R^ and TCATd^R^ isolates show a higher correlation to each other than to the TMP^R^ isolate (Figure 3C,D). This suggests a different gene expression profile or, in other words, a different approach towards resistance development.

### 2.5. Shared Gene Expression between Isolates Corresponds with a Stress Response and Reduced Cell Growth

**Figure 4:**
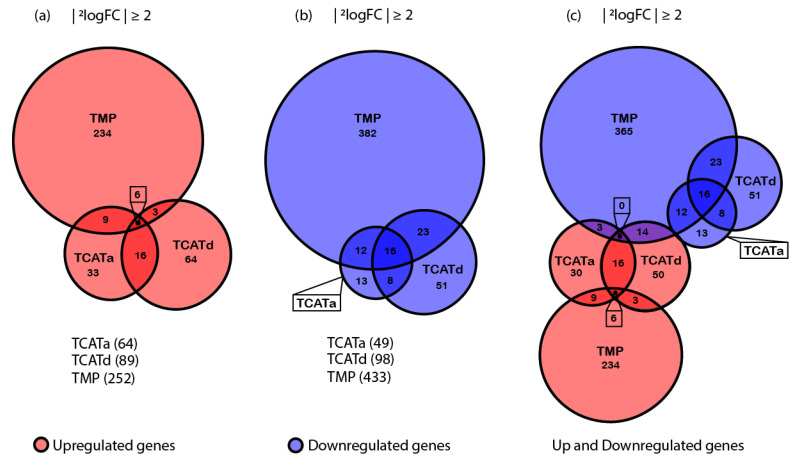
Euler diagrams of differentially expressed genes (DEGs) for each contrast, created with RStudio. Diagrams show how many genes were significantly up- or downregulated (red and blue respectively) and the total number of DEGs using a cutoff fold change of ≥2 and a *p*-value of ≤0.05, respectively: (**a**) upregulated genes; (**b**) downregulated genes; and (**c**) up- and downregulated genes for all contrasts.

To analyze the gene expression, a fold change cutoff of ≥ 2 and *p*-value ≤ 0.05 were used. The TopHits table listing all the differentially expressed genes (Table S1) and an overview of the total number of genes up- and downregulated for each strain compared to the control are shown in Figure 4. 

Differential gene expression analysis revealed that the number of genes affected by resistance development differed for each isolate. Moreover, most genes were affected in the TMP^R^ isolate, where 685 genes undergo a change in expression of >2-fold. Of these genes, 252 were exclusively upregulated and 433 genes were exclusively downregulated. In the TCATa^R^ isolate, there are 64 genes upregulated and 49 genes downregulated, meaning expression for a total of 113 genes was altered, significantly less than in the TMP^R^ strain. In addition, in the TCATd^R^ isolate, the total number of genes affected (187) was lower compared to the TMP^R^ isolate, of which in this case 89 genes were upregulated and 98 genes were downregulated. 

When comparing differential gene expression using Euler diagrams, overlapping genes between samples can be found more easily. TopHits from each contrast were grouped and evaluated in more detail using Gene Set Enrichment Analysis (GSEA-Pro v3.0) to add organism-specific information to the dataset [29]. A subset of genes is similarly expressed in all contrasts (Figure 4). Of the upregulated genes, six genes are shared between all samples. Within this subset, three genes are encoded by the e14 phage-like element and include *yfmN*, *yfmP* and *yfmR*. Upregulation of these genes is thought to play a role in increased acid resistance, increased biofilm formation and reduced cell growth. In addition, the antibiotic mitomycin C was previously reported to convert the e14 cryptic prophage from its lysogenic to its lytic form, increasing its excision rate significantly. A similar response was found upon increased oxidative stress [30,31]. In addition, *yafO*, *umuD* and *torD* are also upregulated in all three isolates. These genes have been reported to inhibit cell growth, follow induction of the SOS response and be involved in anaerobic respiration of trimethylamine N-oxide (TMAO), respectively [32,33,34]. Among the 16 shared downregulated genes, a trend can be seen in the involvement in carbohydrate and coenzyme transport and metabolism, as predicted by GSEA-Pro. Moreover, there is another small set of genes that overlap between different isolates (Figure 4). Closer inspection and evaluation of these gene sets reveal that the gene expression profiles of the TCATa^R^ and TCATd^R^ are more similar to each other than each of them to the TMP^R^ isolate. All overlapping genes between TCATa^R^ and TCATd^R^, TCATa^R^ and TMP^R^ and TCATd^R^ and TMP^R^ are, although not always significant, similarly expressed in all isolates.

### 2.6. TCATa Induces Expression of Colonic Acid, Exopolysaccharides and TolC

In the TCATa^R^ isolate, most upregulated genes are grouped to their involvement in cell wall/membrane/envelope biogenesis by GSEA-Pro. In more detail, the *wza* and *wzc* operons are upregulated and are responsible for the assembly of *E. coli* capsules and exopolysaccharides [35]. The genes *gmd* and *fcl* also belong to this colanic acid biosynthesis gene cluster [36]. Moreover, a previous study has predicted *wza* to function as an efflux protein comparable to *tolC* [37]. Next to this, several genes (*intE*, *ymfH*, *ymfL*, *stfE* and *croE*) of the e14 cryptic prophage and genes related to stress and reduced cell growth (*gadA*, *tisB*, *iraP*, etc.) were upregulated [30,38,39,40]. Indeed, it has been shown before that cryptic prophages can contribute to resistance to sub-lethal concentrations of quinolone and β-lactam antibiotics [31]. Interestingly, *tolC* is also upregulated >3-fold in this strain. Although TolC is known to interact with several efflux pump proteins located in the inner membrane, e.g., ArcA/B, none of these reported interactants are upregulated. Similar to TolC, the protein CusC also belongs to the family of outer membrane efflux proteins. Similar to TolC, CusC interacts with CusB/A to facilitate efflux of mainly copper and silver ions. The *cus* operon also includes a chaperone *cusF* which binds Ag(I) and Cu(I) ions in the periplasmic space and transfers them to CusB for transport [26,41]. Of the *cus* operon, only expression of *cusF* is upregulated in this strain, provoking the thought that TCATa mostly resides in the periplasmic space.

Regarding the downregulated genes, 13 are specific for the TCATa^R^ isolate. The function of several of these genes is not known, but two interesting observations can be made. The gene *sieB* shows similarity to the *dicABCF* region of a defective lambda prophage, which encodes a function responsible for superinfection exclusion [42]. Downregulation of this gene is interesting since many e14 prophage genes are upregulated in this strain and superinfection exclusion confers the infected host with resistance against secondary phage infections. Unfortunately, the mechanisms underlying superinfection exclusion and the fitness consequences for the host are still poorly understood [43]. Secondly, the multidrug resistance protein MdtB is downregulated. This protein is part of the MdtABC complex, a RND-type drug exporter complex that requires TolC to confer a resistant phenotype. Upregulation of one of the *mdt* (A–C) genes is not sufficient to gain resistance, as previously described by Nagakubo and coworkers [44]. Therefore, the effect of sole downregulation of *mdtB* remains unclear.

### 2.7. TCATd Activates Carnitine Synthesis, Maltose Transport and Spermidine Exporters

Many of the genes exclusively upregulated in the TCATd^R^ isolate are related to cell metabolism according to GSEA-Pro analysis. Including Clusters of Orthologous Groups of proteins (COG) in energy production and conversion, amino acid transport and metabolism, carbohydrate transport and metabolism and inorganic ion transport and metabolism. In particular, most predominant upregulated genes are involved in the carnitine metabolism, maltose and maltodextrin transport. The carnitine metabolism has been extensively studied and in prokaryotes carnitine is known as a compatible solute for stress protection, providing improved osmotolerance, thermotolerance, cryotolerance and barotolerance [45]. Furthermore, a recent study investigated the potential of maltose/maltodextrin transport in antibiotic uptake by creating maltodextrin–fluorophore conjugates. Here, it was reported that these conjugates were indeed able to cross both the outer and inner membrane of *E. coli*. Transport across the outer membrane is facilitated by *lamB*, which shows a >3-fold increase in gene expression in the TCATd^R^ isolate, possibly taking over the function of TolC. Low cytoplasmic concentrations were proposed to be an effect of poor transport across the inner membrane, internal degradation or an expulsion via efflux pumps such as AcrAB-TolC. It was noted that cytoplasmic concentrations of the conjugate are able to trigger activation of the maltose regulon, also upregulated in the TCATd^R^ strain [46]. In addition to the many genes upregulated in the two pathways mentioned above, four other genes that could explain the increased resistance were found: *mdaB*, *mqsR*, *mdtI* and *mdtJ*. Overexpression of these genes all result in an increased tolerance to certain, although not the same, antibiotics [47,48]. 

The 51 genes that are specifically downregulated in the TCATd^R^ isolate show a trend in their involvement in thiamine and enterobactin synthesis as well as iron homeostasis, iron import and iron-sulfur cluster assembly. The enterobactin operon is repressed by Fur, a ferric uptake regulator, in iron-rich conditions, while it has been reported to be upregulated by iron limitation and oxidative stress. Downregulation of this operon renders the cells susceptible towards oxidative damage [49]. Enterobactin synthesis proteins are thought to be membrane associated and the export of enterobactin is TolC dependent [50]. Interestingly, several genes related to enterobactin transport and iron uptake (*fhuE*, *fepA* and *cirA*) are TonB-dependent receptors. TonB-dependent receptors mediate substrate-specific transport across the outer membrane including siderophores, vitamin B12, saccharides and aromatic compounds [51,52]. To date, little is published on the effect of downregulation of TonB-dependent receptors on the cell. 

### 2.8. TMP Resistance Is Based on Reduced Cell Growth and Persister Formation

The number of genes differentially expressed (≥2-fold) was significantly higher in the TMP^R^ isolate. The large number of genes affected makes it more difficult to pinpoint the exact consequences of this expression pattern to the cell. Upon treatment with TMP, 234 genes were specifically upregulated in this strain. Again, most upregulated genes were associated with metabolism. In this case, energy production and conversion, amino acid transport and metabolism, nucleotide transport and metabolism, carbohydrate transport and metabolism, lipid transport and metabolism and inorganic ion transport and metabolism. Compared to the other two isolates, here we can see up and downregulation of at least nine transcription factors (for each), potentially explaining the large number of differentially expressed genes. Multiple transcription factors belong to the LysR family (*perK*, *yiaU* and *gadE*), a well characterized group of transcriptional regulators regulating a diverse set of genes [53]. Many of the upregulated genes are normally expressed at the end of the logarithmic growth phase and/or at the beginning of the stationary phase as well as under anaerobic growth conditions (e.g., *appB/C* and *feoA/B)* [54,55]. Specifically, the genes *hipB*, *relE* and *relB* are characteristic for persisters, cells that neither grow nor die in the presence of high concentrations of antimicrobials, and exhibit multidrug tolerance [56]. Together, the expression of these genes is an indication of reduced cell growth. Finally, a large group of genes (75) was predicted to be associated with the plasma membrane, of which a significant part could be identified as transporters, e.g., *modB/C*, *fepD/G*, *nikB/C/D/E*, *adiA/C*, *gadB/C*, *gspE/G/H/K*, *exbB/D*, *araE*, *mdtM/F* and *ybhF/S* [57]. Some of these belong to the MFS family and some are also designated as multidrug transporters [58].

In total, 382 genes specific to the TMP^R^ isolate were downregulated. When running GSEA-pro on this gene set, no significant correlation could be found between the genes. However, it is still worth highlighting some of the downregulated genes. For example, of some operons, multiple genes were downregulated, most of which are involved in the general cell metabolism and in amino acid biosynthesis and metabolism. It was also noted that many of these operons were (in)directly regulated by Fur and/or FNR [55,59]. Although *fur* is also downregulated in the TMP^R^ isolate, expression of *fnr* was not significantly altered. In addition downregulation of many ribosomal proteins was observed, which was previously reported to correlate to reduced cell growth and intracellular (p)ppGpp concentrations [60]. Increased levels of (p)ppGpp also correspond to the downregulation of several other genes involved in metabolism and cell motility. Overall, this expression pattern hints towards reduced cell growth and increased persister formation [61]. Surprisingly, a set of genes encoding transporters such as *chaA* and *lamB* was also downregulated [46,62]. While *lamB* is downregulated in the TMP^R^ isolate, it is upregulated in TCATd^R^. Another contradiction is the downregulation of *wcaE/F*, where *wcaE* is significantly upregulated in the TCATa^R^ isolate. Indicating differences in the mechanism of the acquired resistance between isolates.

**Figure 5:**
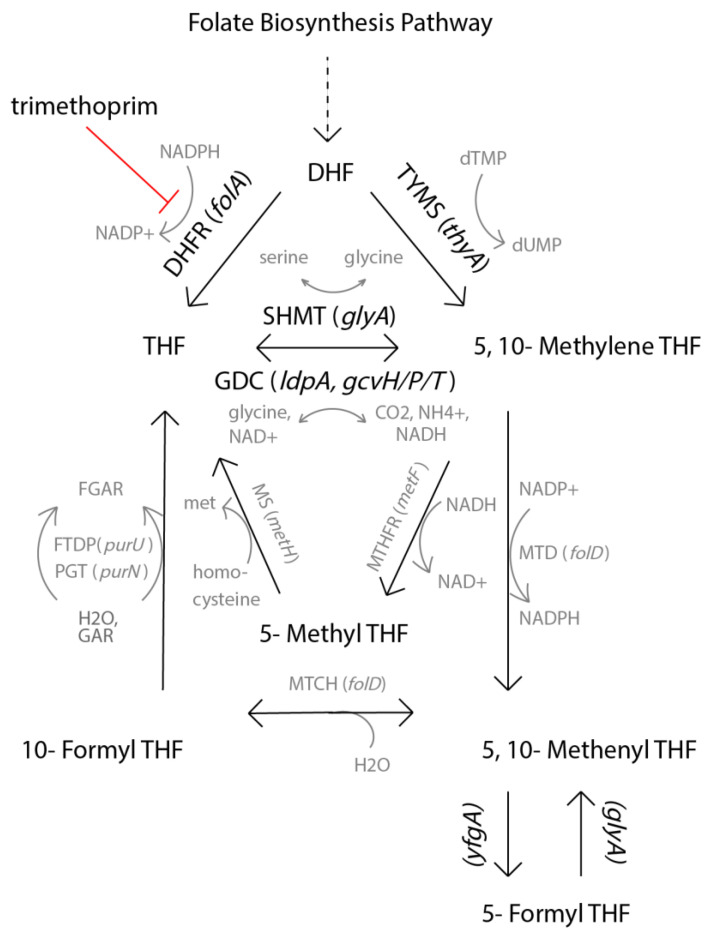
Simplification of the folate biosynthesis pathway. Adapted from Schober et al., *Cell Reports* (2019). Both enzymes and metabolites are abbreviated: dihydrofolate (DHF), tetrahydrofolate (THF), dihydrofolate reductase (DHFR), thymidylate synthase (TYMS), serine hydroxymethyltransferase (SHMT), glycine cleavage system (GDC). Black text and arrows indicate the steps most important in this genetic context. Genes *glyA* and *gcvH/P/T* are significantly downregulated, while *ygfA* is significantly upregulated.

Notably, several genes involved in the folate biosynthesis pathway were differentially expressed. The genes *folE/K*, *queD*, *glyA* and *gcvH/P/T* were downregulated while *ygfA* was upregulated. These data indicate that a different pathway towards the synthesis of tetrahydrofolate (THF) might be favored, reducing dependence on *folA*, the target of TMP (Figure 5) [63].

## 3. Discussion

AMR can be defined as a state in which cells no longer respond to a certain concentration of compound to which they were sensitive before. When cells are grown in the presence of a sub-lethal concentration of an antimicrobial, which is slowly increased over time, they can be driven towards resistance development [3]. The natural variation in sensitivity of different *E. coli* strains towards TMP is considerable and can be dependent on the genetic background of the strain. Resistance to TMP can be mediated through several different mechanisms, for example changes in the permeability barrier and/or efflux pumps, regulational changes in the target enzymes and mutational or recombinational changes in the target enzymes. Mutational changes in the *dhfr* gene can increase the original MIC value over a 1000-fold [19]. The TMP^R^ isolate produced in this study acquired a MIC for TMP that was 25 times greater than the original MIC value of the parent strain. Although this increase in MIC value is significant, the upper limit of resistance was not reached. In the sensitivity of different *E. coli* strains towards the TCAT conjugates as well, a great variation was found (unpublished data). TCAT conjugates are poorly soluble in aqueous solutions, and, upon increasing the concentrations during growth, a breaking point was reached around 98.5 µM, where the compound precipitated out of the solution. For this reason, the MIC value of the thermally adapted (and lowly active) TCATd compound could not be significantly increased. The MIC value against TCATa (highly active) could be increased 10-fold. Due to the difference in antimicrobial activity between the TCATa (on-state) and the TCATd (off-state) conjugates, a significant increase in the MIC value of the parent strain towards the TCATa conjugate was still allowed for, in contrast to TCATd.

To interpret the data obtained from sequencing, it is important to keep the genetic background of the parent strain in mind. The parent strain used here, CS1562, contains a stable Tn10 transposon that is integrated in the *tolC* gene locus. This transposon confers tetracycline resistance, but the location of insertion renders the TolC efflux pump not functional [25,64]. Usually, this strain is grown in the presence of tetracycline (10–15 µg/mL) as a selective pressure to ensure that the transposon is not lost. In this study, however, all samples were grown in the absence of tetracycline so that intrinsic resistance of the cells could develop freely. Genome sequencing revealed that the Tn10 transposon was only lost in the TCATa^R^ isolate. Clean excision of the transposon restored the wild-type genetic sequence of *tolC* and thereby its function. Interestingly, evidence from RNA sequencing also proved TolC to be significantly overexpressed solely in this isolate. In addition to the upregulation of *tolC*, several genes involved in capsule and exopolysaccharide formation are upregulated, possibly as an attempt to hamper entrance of TCATa into the cell [35]. Downregulation of *mdtB* in this isolate is interesting, in the sense that this is an inner membrane drug transporter only able to confer a resistant phenotype in the presence of TolC [44]. The combination of upregulation of an outer membrane efflux pump and exopolysaccharides, and the downregulation of an inner membrane drug exporter, suggests that the TCATa conjugate mostly resides in the periplasmic space of the cells. By altering gene expression in this way, resistance most likely relies on preventing TCATa from entering the cytoplasm and reaching its biological target.

In the TCATd^R^ isolate, the Tn10 transposon is not lost, retaining the loss of function of TolC. In this isolate, we see upregulation of *lamB*, that enables maltose uptake across the outer membrane. Dumont and co-workers have previously described a potential role of the maltose regulon in antibiotic uptake through this maltoporin [46]. Although their investigation focused on the uptake of maltodextrin-fluorophore conjugates and *lamB* is a sugar-specific porin, upregulation of alternative porins, like *lamB* has been reported to balance the loss of general porins, like TolC [46,65]. In addition the *mdtI/J* genes were upregulated, which belong to the small multidrug resistance(SMR) protein family [48]. Possibly, the upregulation of these genes enables the cells to compensate the lack of TolC. In addition, carnitine metabolism, also upregulated in this isolate, can act as a compatible solute under multiple stress conditions. Although the exact mechanism underlying this protection is not yet fully understood, it is known to play an important role in cell viability and proliferation [45]. In combination with the downregulation of the enterobactin operon, which renders the cells sensitive to oxidative damage, this indicates activation of a stress response. Furthermore, enterobactin transport is TolC-dependent and the lack of functional TolC in this isolate might also indirectly downregulate the expression of this operon to reduce cell envelope stress [50]. Overall, this gene expression profile, although not the same, is mechanistically very similar to the effect of the altered gene expression in the TCATa^R^ isolate, suggesting the acquired resistance in the TCATd^R^ isolate also mostly relies on efflux of TCAT from the cell. The TCATd^R^ isolate also did not excise the Tn10 transposon, indicating that the selective pressure of the applied concentration of compound (not significantly increased) might not have been high enough, and the cells were not (yet) driven towards this decision.

In contrast to the TCAT^R^ isolates, the acquired resistance in the TMP^R^ isolate seems to rely mostly on the downregulation of cell metabolism and persister formation. In this isolate, the expression of several transcription factors is altered, affecting a large set of genes. Many of these genes indicate a reduction in cell metabolism, cell growth and mobility. These characteristics are also accounted for in persisters. Persisters are genetically identical to the wild type cells but present a dormant, non-dividing phenotype that exhibits multidrug tolerance and can survive treatment of high concentrations of antimicrobials. On top of the general reduction in cell metabolism, a set of genes, e.g., *hipA* and *relE*, specifically upregulated in persister cells was also found [56]. It has been reported that ectopic expression of these genes induces a state of reversible dormancy and produces a multidrug tolerant state that mimics naturally formed persisters. The multidrug tolerance exhibited by persisters is mechanistically distinct from genetically acquired resistance and not well-understood. It appears that, in a dormant state, the activity of antibiotic targets is diminished. By reducing metabolism of the cells, persisters reduce activity of the molecules targeted by the antibiotics. It is thought that in this way the antibiotics are not prevented from binding their target molecule, but are unable to corrupt their cellular function, accounting for the increased tolerance [66]. In addition, regulation of certain genes involved in the folate biosynthesis was changed. Although expression of DHFR, the target of TMP, was not altered, expression of *folE*, *folK*, *gvcH/P/T*, *glyA* and *queD* was downregulated while the expression of *ygfA* was upregulated. These changes suggest that a different path towards the production of THF is favored, bypassing the role of DHFR in the reduction of DHF to THF [63]. The gene expression profile of the TMP^R^ isolate also showed some contradictions to the other two isolates in the downregulation of *lamB* (up in TCATd^R^) and *wcaE/F* (up in TCATa^R^), reinforcing the idea that a different approach is taken towards resistance development. Alteration of the cell metabolism and folate biosynthesis also suggests TMP is indeed able to enter the cytoplasm of the cell and reach its target, whereas resistance towards the TCAT conjugates is based on the prevention of the conjugates to enter into the cytoplasm of the cells.

In vitro bioactivity assays reveal that TCAT is a potent inhibitor of DHFR both before and after irradiation with λ = 627 nm light. On the other hand, gene expression analysis in this study shows that in vivo resistance to TMP and the TCAT conjugates is acquired differently. Resistance to the TCAT conjugates depends on limiting the entrance of the molecule into the cell, while resistance to TMP is mainly acquired by persister formation. 

## 4. Materials and Methods

### 4.1. Strains and Culture Conditions

*E. coli* CS1562 (*tolC6:tn10*) was used as the background strain for all experiments. This strain has a stable Tn10 transposon conferring resistance against tetracycline that is inserted in the *tolC* gene locus [25]. The strain was grown in cation adjusted Mueller–Hinton broth (MHB II, Sigma Aldrich) at 37 °C, 220 rpm and was always protected from light by tin foil, unless otherwise stated. MHB II broth was supplemented with 1.7% agar (Boom) and the respective final concentration of antimicrobial (TMP 1.4 µM, TCATa 49.3 µM, TCATd 98.5 µM) to provide a solid base to assess colony uniformity before selection of the final isolates. For −80 °C stocks, a final concentration of 15% glycerol was taken and the respective antimicrobial was always present. For the isolates, single colonies were picked and enriched by a final round of growth in medium containing their (highest) respective antimicrobial before DNA and RNA isolation was performed.

### 4.2. Preparation of TCAT Solutions

The synthesis and the photochemical characterization of TCAT was previously reported [12]. A 16.4 mM stock solution of TCAT in DMSO was divided into two parts. One half was thermally adapted with a heat gun for 1 min to obtain only TCATd. To reach the photostationary state (PSS) with a 87:13 *cis:trans* distribution, the second half of the stock solution was irradiated for 3 h with a 627 nm LED, in a 10 mm quartz cuvette under constant stirring.

### 4.3. Protein and DNA Sequences

>WT-DHFR-_His_ (C85A, C152S) [67] (Protein sequence)

MAHHHHHHGSAMISLIAALAVDRVIGMENAMPWNLPADLAWFKRNTLDKPVIMGRHTWESIGRPLPGRKNIILSSQPGTDDRVTWVKSVDEAIAAAGDVPEIMVIGGGRVYEQFLPKAQKLYLTHIDAEVEGDTHFPDYEPDDWESVFSEFHDADAQNSHSYSFEILERR

>WT-DHFR-_His_ (C85A, C152S) [67] (DNA sequence)

ATGGCTCACCACCACCACCACCACGGTTCGGCTATGATTTCTCTGATTGCGGCACTGGCTGTCGATCGTGTTATTGGTATGGAAAACGCTATGCCGTGGAATCTGCCGGCTGATCTGGCGTGGTTTAAACGTAACACCCTGGACAAGCCGGTCATTATGGGCCGCCATACGTGGGAAAGCATCGGTCGTCCGCTGCCGGGTCGCAAAAATATTATCCTGAGCAGCCAGCCGGGCACCGATGACCGTGTGACGTGGGTTAAGAGCGTCGATGAAGCAATTGCGGCGGCAGGCGACGTGCCGGAAATTATGGTTATCGGCGGTGGCCGCGTTTATGAACAGTTCCTGCCGAAAGCCCAAAAGCTGTACCTGACCCATATCGATGCAGAAGTCGAAGGTGATACGCACTTTCCGGACTATGAACCGGATGACTGGGAAAGTGTGTTCTCCGAATTTCACGACGCCGACGCTCAGAACAGCCACTCATACTCATTCGAAATCCTGGAACGCCGTTGATAAAAGCTT

### 4.4. Overexpression of E. coli DHFR (DHFR) Protein

The pT7-SC1 plasmid containing the DHFR genes were transformed into *E. cloni*^®^ EXPRESS BL21 (DE3) cells (Lucigen), and transformants were selected on LB agar plates supplemented with 100 µg/mL ampicillin after overnight growth at 37 °C. The resulting colonies were grown at 37 °C in 2xYT medium supplemented with 100 µg/mL ampicillin until the optical density at 600 nm (OD_600_) reached ~0.8 (200 rpm shaking). The DHFR expression was subsequently induced by addition of 0.5 mM IPTG (isopropyl β-D-1-thiogalactopyranoside), and the temperature was switched to 25 °C for overnight growth (200 rpm shaking). The next day the bacteria were harvested by centrifugation at 6000× *g* at 4 °C for 25 min and the resulting pellets were frozen at −80 °C until further use.

### 4.5. Purification of DHFR Protein 

Bacterial pellets originating from 50 mL culture were resuspended in 30 mL lysis buffer (150 mM NaCl, 15 mM Tris-HCl pH 7.5, 1 mM MgCl_2_, 0.2 units/mL DNaseI, 10 µg/mL lysozyme) and incubated at 37 °C for 20 min. After further disruption of the bacteria by probe sonication the crude lysate was clarified by centrifugation at 6000× *g* at 4 °C for 30 min. After lysis of the bacteria by probe sonication, the crude lysates were clarified by centrifugation at 6000× *g* for 20 min at 4 °C and the supernatant was mixed with 200 µL of Ni-NTA resin (Qiagen) equilibrated in wash buffer. After 1 h, the resin was loaded into a column (Micro Bio Spin, Bio-Rad) and washed with ~5 mL of the wash buffer. DHFR was eluted with approximately ~0.5 mL of wash buffer containing 500 mM Ethylenediaminetetraacetic acid (EDTA).

### 4.6. E. coli DHFR Inhibition Assay

TCAT and TMP were tested as DHFR inhibitors via a colorimetric assay (Sigma-Aldrich, Catalog No. CS0340). The stock solutions of the inhibitors were prepared in DMSO (1 mM). Thermal adaptation and irradiation of TCAT samples were carried out as described in “Preparation of TCAT solutions” section. Instead of the human DHFR protein provided in the kit, the in-house purified DHFR was used. In a 3 mL cuvette, DHFR (~20 ng) was incubated with NADPH (60 µM, final) for 3 min, followed by an incubation with the inhibitor (from 10^−5^ to 10^−12^ M, final) for additional 7 min. After the UV–Vis spectrophotometer (Agilent 8453) was blanked with this solution, dihydrofolic acid (15 µM, final) was added and the absorbance at 340 nm was monitored every 5 s for 300 s at 25 °C. The concentration of DMSO in the final volume was 1% *v*/*v* for all enzymatic reactions. The measurements were performed in triplicates. For the control enzymatic reactions, DHFR was incubated with NADPH and DMSO (1 % *v*/*v*, final). Linear regression was performed on Microsoft Excel to calculate ΔAbs/s. % DHFR activity was calculated as the ratio between ΔAbs/s for the sample and ΔAbs/s for the control reaction. GraphPad Prism 5.0 (GraphPad Software, Inc., version 5.00) was used for the determination of the IC_50_ of each inhibitor. Nonlinear regression was used for data fitting.

### 4.7. Minimal Inhibitory Concentration (MIC) Determination

All MIC values were conducted in at least three biological and three technical replicates according to the CLSI standards [68].

### 4.8. Generation of Resistant Strains

Stock solutions were prepared by dissolving either TMP (Sigma Aldrich, Darmstadt, Germany) or TCAT conjugates in DMSO and were used to obtain resistant isogenic variants of *E. coli* CS1562. TCAT conjugates were always radiated before use, as previously described in this article, to ensure maximal conversion towards the TCATa or TCATd conjugate. Resistant variants were generated via the following procedure, adapted from a similar method described before [3]; the strains were grown in MHB II broth with 0.013 µM TMP, 1.6 µM TCATa and 9 µM TCATd, respectively, which is well below the respective MIC values. Subsequently, the strains were repeatedly inoculated in media with increasing concentrations of the respective antimicrobial, either TMP, TCATa or TCATd. A control sample repeatedly inoculated without the addition of any compounds was also taken along. After every 8th hour of growth, OD_600_ measurements were taken and resistant cultures were complimented with glycerol, snap-frozen in liquid nitrogen and stored at −80 °C until the next stepwise increment. The resistant cultures were plated on antimicrobial-containing plates and single colonies were picked for further enrichment and sequencing purposes. The respective antimicrobial, i.e., TMP, TCATa or TCATd, was always added to the growth media to maintain resistance.

### 4.9. Genomic DNA Isolation and Sequencing

Enriched cultures were re-grown in media containing 1.4 µM TMP, 49.3 µM TCATa, 98.5 µM TCATd or without addition of compounds for the control respectively, for 6 h, after which the cells were harvested by centrifugation. Genomic DNA was isolated using the GenElute™ Bacterial Genomic DNA kit (NA2110, Sigma Aldrich, Darmstadt, Germany). Isolated genomic DNA was sent to BGI (Wuhan, China) for WGS on DNBseq™. 

A reference genome was created from the raw sequence reads of the propagated parent strain using Bowtie, version 2.3.4.3-foss-2018a [27]. Raw sequence reads from all other isolates were compared to this reference using breseq, version 0.35.4-foss-2020a-R-4.0.0 to look for genomic mutations [69]. This Whole Genome Shotgun project has been deposited at DDBJ/ENA/GenBank under the accession JAGEPG000000000. The version described in this paper is version JAGEPG010000000. 

### 4.10. Total RNA Isolation and Sequencing

Enriched cultures were re-grown in media containing 1.4 µM TMP, 49.3 µM TCATa, 98.5 µM TCATd or without addition of compounds for the control, respectively, for 6 h, after which the cells were harvested by centrifugation. RNA was isolated using a High Pure RNA Isolation Kit (Roche, Basel, Switzerland). 

RNA samples were sequenced at BGI (Wuhan, China), who performed rRNA removal and library preparation. Transcriptome sequencing was performed on DNBseq™. Raw sequence reads were analyzed for quality and mapped to *E. coli* K12 (ASM983882) using Bowtie2 [27]. Count values were used as an input for the T-REx analysis pipeline for statistical analysis to determine differentially expressed genes [28]. For the T-REx analysis, a text file describing the factors, contrasts and classes specifying genes were written. TopHits were further evaluated with GSEA- Pro v. 3 [29]. Euler diagrams were made with RStudio version 1.3.1093. The RNA-seq data were uploaded under GEO accession number: GSE169069.

## 5. Conclusions

The TCAT conjugates reside mostly in the periplasmic space and cells are able to prevent the conjugates from reaching their biological target, DHFR. In response to TMP, resistance is conferred by reducing the metabolic effects of DHFR inhibition by TMP. These findings show the importance of the defense system of Gram-negative bacteria and indicate that, despite the same biological target, the in vivo mechanism of action and/or resistance development can be considerably different.

The results of this study are very meaningful, although it must be mentioned that the parent strain used here (CS1562) is a derivative strain in which one of the major efflux pumps (TolC) is no longer functional. Excision of the Tn10 transposon and restoration of *tolC* in the TCATa^R^ isolate indicates that TolC plays a significant role in the sensitivity of the cells to these conjugates. Several other *E. coli* strains were also initially assessed for their MIC values against the TCAT conjugates and included *E. coli* ATCC35218 and the general lab strains *E. coli* Top10 and *E. coli* Dh5α (unpublished data). In these strains, the MIC values were already over 246.3 µM for the active TCATa conjugate and deemed irrelevant due to the solubility issues of the conjugates in aqueous solution. The results obtained must therefore be viewed as a proof of principle and will have limited clinical relevance, because in therapeutic treatment administration of such high concentrations of TCATa is challenging and the pathogenic strain is most likely not sensitive to this particular compound. However, these results do provide a better understanding in the development of new therapeutic strategies such as photopharmacology and its consequences on resistance development, and the development of new photoactivatable antibiotics with higher intrinsic killing activity is awaited.

## Figures and Tables

**Table 1:** Minimal inhibitory concentration for TMP, TCATa and TCATd of the *E. coli* parent strain and resistant isolates. As well as the number of generations grown during the experiment. Resistant isolates from each condition are characterized by a superscript R (^R^).

Strain	MIC TMP (µM)	MIC TCATa (µM)	MIC TCATd (µM)	Number of Generations
*E. coli* CS1562	0.05	4.9	80.1	32
TMP^R^	1.4			21
TCATa^R^		49.3		23
TCATd^R^			98.5	12

**Table 2:** Mutations shared between all resistant isolates. Arrows (→) in the first column (Mutation) indicate the nature of the mutation, for example, G→A indicates guanine is replaced by adenine. Deletions are denoted by the delta sign (Δ). Addition of an amino acid is indicated by a plus sign (+). The arrows in the column Gene indicate the direction of transcription of the genes on the genome and intergenic regions are denoted by a forward slash (→/←). In other words, the mutation G→A occurs in the intergenic region (/) between the genes *aqpZ* and *lysO* which are both forwardly transcribed (→) on the genome. Evidence from mapped reads is categorized in read alignment (RA), missing coverage (MC) and/or new junction (JC).

Predicted Mutations						
Mutation	Annotation	Gene	Description	Evidence	Seq Id	Position
**G** **→** **A**	Intergenic (+202/−293)	*aqpZ→*/*→lysO*	aquaporin Z/L lysine exporter LysO	RA	NODE_2	134,755
**Δ** **2bp**	Intergenic (+2/−2)	*Ecoli_02842→*/*→Ecoli_02843*	tRNA Val/tRNA Lys	RA	NODE_20	45,821
**Δ** **1bp**	Intergenic (+3/−2)	*Ecoli_02844→*/*→Ecoli_02845*	tRNA Val/tRNA Lys	RA	NODE_20	46,127
**A** **→** **G**	Intergenic (+1023/+707)	*chaA→*/*←kdsA*	sodium potassium/proton antiporter ChaA/3 deoxy 8 phosphooctulonate synthase	RA	NODE_4	17,237
**A** **→** **C**	Intergenic (+1387/+343)	*chaA→*/*←kdsA*	sodium potassium/proton antiporter ChaA/3 deoxy 8 phosphooctulonate synthase	RA	NODE_4	17,601
**Δ** **1bp**	Intergenic (+1420/+310)	*chaA→*/*←kdsA*	sodium potassium/proton antiporter ChaA/3 deoxy 8 phosphooctulonate synthase	RA	NODE_4	17,634
**+A**	Intergenic (+1425/+305)	*chaA→*/*←kdsA*	sodium potassium/proton antiporter ChaA/3 deoxy 8 phosphooctulonate synthase	RA	NODE_4	17,639
**C** **→** **A**	Intergenic (+1428/+302)	*chaA→*/*←kdsA*	sodium potassium/proton antiporter ChaA/3 deoxy 8 phosphooctulonate synthase	RA	NODE_4	17,642
**T** **→** **C**	Intergenic (−/−)	−/−	−/−	RA	NODE_97	18
**T** **→** **G**	Intergenic (−/−)	−/−	−/−	RA	NODE_97	65
**G** **→** **A**	Intergenic (−/−)	−/−	−/−	RA	NODE_97	98
**C** **→** **T**	Intergenic (−/−)	−/−	−/−	RA	NODE_97	104
**G** **→** **T**	*L210L (CTG* *→* *CTT)*	Ecoli_03722 →	IS5 like element ISKpn26 family transposase	RA	NODE_39	4254

**Table 3:** Genomic mutations specific for the TCATa^R^ isolate. Deletions are denoted by the delta sign (Δ). Evidence from mapped reads is categorized in read alignment (RA), missing coverage (MC) and/or new junction (JC).

Predicted Mutations						
**Mutation**	Annotation	Gene	Description	Evidence	Seq Id	Position
**Δ** **1bp**	coding (87/1257 nt)	*Ecoli_01894 →*	MFS transporter	RA	NODE_10	108,811
**Δ2934 bp**		*Ecoli_03163 [tolC_2]*	*Ecoli_03163, tetR(A), tet(A), tetC, [tolC_2]*	MC, JC *→*	NODE_26	7555

**Table 4:** (**A**) File describing the experiment factors. (**B**) File describing the comparisons made during the analysis of differential gene expression. Resistant isolates from each condition are characterized by a superscript R (^R^).

(A) Factors		(B) Contrasts
Experiment	Strain	
C	*E. coli CS1562* (propagated parent strain as a control)	
TCATa^R^	TCATa resistant isolate	TCATa^R^-C
TCATd^R^	TCATd resistant isolate	TCATd^R^-C
TMP^R^	TMP- resistant isolate	TMP^R^-C

## Data Availability

The sequencing data presented in this study are openly available in Table S1, online at NCBI and can be found here: [GSE169069, https://www.ncbi.nlm.nih.gov/geo/query/acc.cgi?acc=GSE169069, accessed on 18 March 2021], [JAGEPG000000000, https://www.ncbi.nlm.nih.gov/nuccore/JAGEPG000000000, accessed on 17 March 2021]. Unpublished data referred to in this study are available on request from the corresponding author.

## Supplementary Materials

The following are available online at https://www.mdpi.com/article/10.3390/ph14050392/s1, Table S1: Differentially expressed genes. The differentially expressed genes for all contrasts are listed, using a cutoff fold change of ≥2 and *p*-value ≤ 0.05.
